# Validation of a Patient–Reported Outcome Measure in Parkinson’s Disease

**DOI:** 10.3390/nu18071118

**Published:** 2026-03-31

**Authors:** Laurie K. Mischley, Magdalena Murawska

**Affiliations:** 1 Research Institute, School of Naturopathic Medicine, Bastyr University, Kenmore, WA 98028, USA; 2 Department of Radiology (Research), School of Medicine, University of Washington, Seattle, WA 98109, USA; 3 Parkinson Center for Pragmatic Research, Seattle, WA 98133, USA; mmurawska@biostatcode.org

**Keywords:** remote patient monitoring, disease-modification, neuroprotection, rating scales, quality of life, scale responsiveness, measurement sensitivity, clinically meaningful change, pragmatic research, patient-centered research

## Abstract

Background: The Patient–Reported Outcomes in Parkinson’s Disease (PRO-PD) scale is a 35-item visual analog measure designed to quantify symptom severity across motor and non-motor domains. Developed as a continuous, patient-centered outcome, PRO-PD captures patient-perceived change over time and is suitable for remote longitudinal assessment. This study evaluated the psychometric properties of PRO-PD across two independent datasets, including reliability, validity, factor structure, and minimal clinically important difference (MCID), and assessed its relevance to nutrition- and lifestyle-focused research. Methods: Convergent validity was evaluated in a cross-sectional clinical dataset (*n* = 46) using established clinician-rated and patient–reported instruments, including Hoehn and Yahr, Unified Parkinson’s Disease Rating Scale (UPDRS), Parkinson’s Disease Questionnaire-39 (PDQ-39), Montreal Cognitive Assessment (MoCA), and PROMIS measures. Internal consistency, temporal stability, factor structure, and known-groups validity were assessed in a large remote-monitoring cohort (*n* = 2612). MCID thresholds were estimated in a longitudinal subsample (*n* = 390) using anchor-based methods, multinomial regression, and receiver operating characteristic analyses. Results: PRO-PD demonstrated strong convergent validity with established clinical measures, excellent internal consistency (Cronbach’s α = 0.93 (95% CI: 0.90–0.96) for Small Data and 0.95 (95% CI: 0.947–0.951) for Big Data”), and good test–retest reliability (ICC = 0.78 overall; 0.89 at 6 months). Confirmatory testing of a previously proposed eight-factor structure showed suboptimal fit, leading to a parsimonious four-factor solution (Neurobehavioral, Autonomic, Motor, Mood/Motivation) explaining 47.6% of variance. PRO-PD scores increased significantly with advancing disease duration and stage. MCID thresholds were +53.5 points for worsening and −78.5 points for improvement (AUC = 0.64 for worsened vs. not worsened; AUC = 0.71 for improved vs. worsened), with greater sensitivity for detecting deterioration than improvement. PRO-PD scores demonstrated sensitivity to patient-perceived symptom change over time, supporting its utility for longitudinal monitoring and potential application in lifestyle-focused intervention research. Conclusions: These findings support PRO-PD as a psychometrically robust outcome measure that can be completed remotely without trained administrators.

## 1. Introduction

Parkinson’s disease (PD) is estimated to impact over 11 million people worldwide, and the Global Burden of Disease analyses indicate it is among the fastest-growing neurological disorders globally [1,2]. In the United States alone, the economic burden was estimated to be $51.9 million for the 1 million diagnosed individuals in 2017 [3]. Despite extensive research efforts, no established therapies have been shown to slow, halt, or reverse disease progression [4]. Contributing factors include heterogeneity in disease presentation, incomplete understanding of the underlying pathophysiology, and methodological challenges in trial design, including the limited availability of outcome measures that capture early-stage disease burden [2].

Traditional PD severity scales, including clinician-rated motor assessments and stage-based classifications, rely heavily on observable motor features that emerge later in the disease course. The Movement Disorders Society- Unified Parkinson’s Disease Rating Scale (MDS-UPDRS) [5] is the current gold standard in clinical trials [6,7,8], yet currently employed instruments have demonstrated floor effects that limit sensitivity in early PD [9]. Recent expert and stakeholder evaluations have further concluded that traditional clinical outcome assessments, including the MDS-UPDRS, are insufficiently sensitive to early clinical change and day-to-day symptom burden [10]. Although psychometrically robust, the MDS-UPDRS does not provide a global subjective severity rating and may inadequately capture symptom domains that people with PD report as bothersome [11,12]. In concert with biological heterogeneity and incomplete understanding of disease mechanisms, reliance on motor-centric clinician-rated scales represents a methodological challenge that may constrain the detection of gradual, multisystem, and patient-perceived changes expected from many interventions, particularly in early disease.

Nutrition, dietary patterns, and nutraceutical interventions are widely used and actively studied, yet research on these exposures is limited by outcome measures that are often insensitive to gradual, patient-perceived change and poorly suited to remote longitudinal studies. This is particularly problematic for nutrition-based interventions, whose effects are predicted to be modest and cumulative. Regulatory agencies have therefore emphasized the use of validated patient–reported outcomes (PROs) [13]. Establishing the validity and responsiveness of the PRO-PD instrument is a necessary step before longitudinal datasets can be meaningfully analyzed in relation to dietary and nutritional exposures, making this work directly relevant to the scope of *Nutrients*.

The Patient–Reported Outcomes in Parkinson’s Disease (PRO-PD) scale was developed as a patient-centered, continuous outcome measure designed to quantify both motor and non-motor symptom severity without reliance on in-clinic assessments. It was designed to be sensitive early in disease, stable across daily fluctuations, and capable of detecting gradual change over time. The scale was constructed iteratively over several weeks of patient care, followed by a review of the existing literature and circulation among individuals with PD and Movement Disorders Specialists, with additional symptoms incorporated through successive rounds of feedback. To minimize respondent burden, simplicity was emphasized throughout development. Design features included the use of a consistently labeled slider bar (left = good, right = bad) and usability testing to ensure successful completion by a 10-year-old child (OA). Individuals are asked to report the average severity of symptoms over the preceding week, a design choice intended to reduce the influence of medication-related daily fluctuations that can confound snapshot assessments, although some difficulty with estimating averages is anticipated for certain individuals. The English version of the scale has been in use since 2013 [14] and subsequently translated and formally validated in Swedish in 2022 [15], and is being employed as an outcome measure in various clinical trials in Sweden, Australia, and the USA, ClinicalTrial ID: NCT05528302, NCT03152721, and NCT02194816, respectively.

Despite its increasing use as a remote patient–reported outcome measure, the psychometric properties of the English version of the PRO-PD scale have not been comprehensively evaluated. The primary objective of this study was therefore to assess the psychometric performance of PRO-PD, including (1) convergent validity with established clinician-rated and patient–reported PD instruments; (2) discriminative (known-groups) validity, defined as the ability to differentiate participants by disease severity and duration; and (3) responsiveness, assessed by examining whether longitudinal changes in PRO-PD correspond to patient-perceived change using anchor-based methods and clinically meaningful thresholds.

## 2. Materials and Methods

### 2.1. Data Sources

#### 2.1.1. MVP Longitudinal Dataset (“Big Data” (*n* = 2612))

The Modifiable Variables in Parkinsonism (MVP) study is an internet-based longitudinal cohort examining dietary, lifestyle, environmental, and psychosocial factors associated with PD outcomes. Participants complete self-reported assessments biannually. Only participants reporting a diagnosis of idiopathic PD at baseline were included in the present analyses; individuals reporting atypical Parkinsonism or Parkinson-plus syndromes were excluded. PRO-PD data included 35 symptom items (including restless legs) collected at multiple time points [13]. The Small Data clinical trial administered 34 items and did not include restless legs.

#### 2.1.2. Clinical Trial Dataset (*n* = 46) “Small Data” (*n* = 46)

Participants with a diagnosis of PD made by a neurologist completed baseline evaluations for a clinical trial in which the PRO-PD was administered as a secondary outcome. Participants completed PRO-PD alongside established clinical and patient–reported measures, including Hoehn and Yahr staging, UPDRS, PDQ-39, MoCA, Non-Motor Symptom Scale, and PROMIS Global Health.

### 2.2. Overview of Study Samples

Sample 1: Convergent validity sample (*n* = 46)—Baseline data obtained from an in-person clinical trial that included traditional outcome measures as well as PRO-PD scores. All study participants reported a diagnosis of idiopathic PD made by a neurologist and underwent comprehensive in-clinic assessments, including UPDRS by an MDS-certified rater. Other outcome measures included Hoehn and Yahr staging, PDQ-39, MoCA, NMSQ, and PROMIS measures at a single visit. This cross-sectional sample was used exclusively to assess convergent validity between PRO-PD and established clinical instruments [16].

Sample 2: Longitudinal cohort (*n* = 2612)—Participants in the MVP observational study who completed PRO-PD assessments remotely at multiple timepoints over 6–24 months. This sample was used to evaluate internal consistency, test–retest reliability (temporal stability), factor structure, and known-groups validity based on disease duration and demographic characteristics. This ongoing study has maintained ongoing approval by the Bastyr University IRB; ClinicalTrials.gov: NCT02194816 [14,17].

Sample 3: Anchor-based responsiveness subsample (*n* = 390)—A subset of the longitudinal cohort (Sample 2) who completed both baseline and 6-month PRO-PD assessments and provided a patient global impression of change rating at 6 months. This subsample was used specifically to determine minimal clinically important difference (MCID) thresholds using anchor-based methods.

Patient-Perceived Global Change Anchor (MVP Cohort): Within the MVP longitudinal cohort, participants completing both baseline and 6-month PRO-PD assessments were additionally asked to report their perceived disease progression over the prior six months (“Improved,” “Been stable,” or “Worsened”). This investigator-designed, single-item global assessment was specified a priori as an external anchor to assess responsiveness and estimate clinically meaningful change. The item was intended to capture the patient’s integrated judgment of change across both motor and non-motor symptoms over a clinically meaningful time horizon. Differences in longitudinal change in PRO-PD scores were therefore examined across self-reported progression categories to evaluate whether changes in PRO-PD corresponded to patient-perceived improvement, stability, or worsening.

Each analysis was conducted using the sample with the relevant data for the psychometric property being evaluated.

PRO-PD Scale: The PRO-PD is a patient–reported outcome measure assessing 35 Parkinson’s disease symptoms using visual analog scales (0–100), with higher scores indicating greater symptom severity. The total PRO-PD score is calculated as the sum of all item scores.

#### 2.2.1. Statistical Analysis

All analyses were performed using R version 4.2.1 and R Studio version 2025.09.1. The significance level α = 0.05 was used throughout. Data are presented as median and interquartile range (IQR) or mean and standard deviation (SD) as appropriate.

#### 2.2.2. Descriptive Statistics and Item Analysis

For both data sets, individual items were assessed for central tendency (mean), standard deviation, skewness, and proportion of minimum (floor) and maximum (ceiling) values. Ceiling effects were defined as >15% responses with maximum values. Corrected item-total correlations were calculated for each item.

#### 2.2.3. Internal Consistency

Internal consistency was evaluated using Cronbach’s α at baseline for both Small Data (34 items) and Big Data (35 items), with values > 0.90 indicating excellent internal consistency.

#### 2.2.4. Temporal Stability (Test–Retest Reliability)

Temporal stability was assessed using intraclass correlation coefficients (ICC) calculated from mixed-effects models with random intercepts for participants (Big Data only). ICC was computed for each PRO-PD item and the total score across all available time points, and separately for the baseline-to-6-month period. Ninety-five percent confidence intervals for item-level ICC were estimated using bootstrap resampling (500 iterations). ICC values were interpreted as: <0.50 (poor), 0.50–0.75 (moderate), 0.75–0.90 (good), and >0.90 (excellent) temporal stability.

#### 2.2.5. Factor Structure

Confirmatory factor analysis (CFA) was performed on Big Data to test the Swedish 8-factor model structure. Model fit was evaluated using: Comparative Fit Index (CFI; acceptable > 0.90, good > 0.95), Tucker–Lewis Index (TLI; acceptable > 0.90, good > 0.95), Root Mean Square Error of Approximation (RMSEA; acceptable < 0.08, good < 0.06), and Standardized Root Mean Square Residual (SRMR; acceptable < 0.08, good < 0.05). When the Swedish 8-factor model showed inadequate fit, exploratory factor analysis (EFA) with varimax rotation was conducted to identify the optimal factor structure. Factor solutions with 4–7 factors were examined. The number of factors to retain was informed by plot (Figure S1), Kaiser criterion (eigenvalue > 1), interpretability, and variance explained.

Parallel analysis compared observed eigenvalues with simulated random data (100 iterations). Factor analysis was not performed on Small Data due to insufficient sample size.

#### 2.2.6. Convergent Validity

Convergent validity was assessed using Pearson’s correlations between PRO-PD total score and established scales (H&Y, MoCA, NMSQ, PDQ-39, UPDRS, PROMIS) in Small Data. Correlation coefficients were interpreted as: weak (0.10–0.39), moderate (0.40–0.69), strong (0.70–0.89), or very strong (0.90–1.00).

#### 2.2.7. Known-Groups Validity

Known-groups validity was evaluated by comparing PRO-PD scores across clinically relevant subgroups using Kruskal–Wallis tests with post hoc pairwise Wilcoxon tests (Holm adjustment). Comparisons included: Disease duration groups (0–2, 3–4, 5+ years since diagnosis), Disease severity by H&Y stage (Mild ≤ 2, Moderate = 3, Severe ≥ 4) (Small Data), and Gender. Relationships between PRO-PD and continuous variables (age, years since diagnosis) were assessed using Pearson’s correlation.

#### 2.2.8. Minimal Clinically Important Difference (MCID)

At the 6-month follow-up assessment, participants in the anchor subsample (*n* = 390) were asked to select the single best response to the statement, “Over the past 6 months, would you say your disease has: Improved, Been Stable, Worsened? This patient–reported anchor classification served as the external criterion against which PRO-PD change scores were evaluated to identify clinically meaningful thresholds. PRO-PD change scores (6-month minus baseline) were compared across anchor groups using: Kruskal–Wallis test with post hoc pairwise Wilcoxon tests (Holm adjustment), Multinomial logistic regression with anchor status as outcome and PRO-PD change as predictor. Receiver operating characteristic (ROC) analysis was used to determine optimal PRO-PD change thresholds using Youden’s index (sensitivity + specificity − 1). ROC analyses were conducted for: Worsened vs. Stable, Improved vs. Stable, Improved vs. Worsened, Worsened vs. Not-Worsened (combined Improved/Stable). Area under the curve (AUC), optimal threshold, sensitivity, specificity, and accuracy were reported for each comparison.

## 3. Results

Available demographic data on study participants is presented in Table 1.

**Figure 1:**
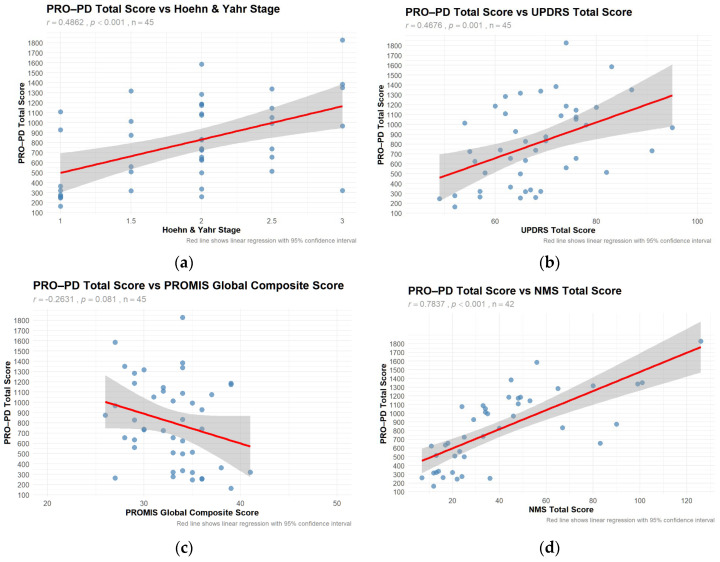

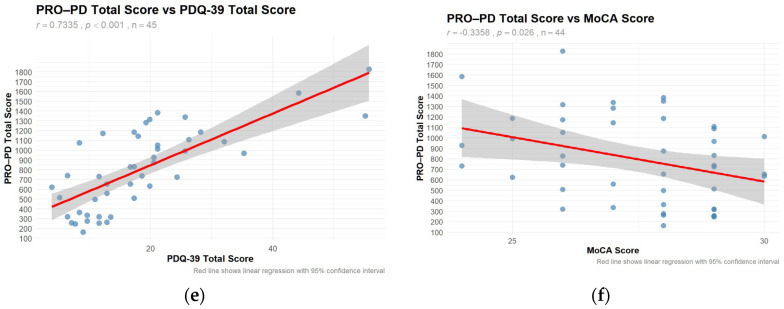
Correlations with Other Scales The 34-item small dataset was used to evaluate convergent validity, which was missing the ‘restless legs’ variable. These figures show the correlation between PRO-PD scores and (**a**) Hoehn & Yahr, (**b**) UPDRS total, (**c**) PROMIS Global quality of life, (**d**) Nonmotor Symptom Scale, (**e**) PDQ-39, and (**f**) MoCA.

Using the PRO-PD scores that had a corresponding in-person clinical evaluation related to a PD research study (*n* = 45), correlation coefficients between PRO-PD and historically used scales [5,13] were calculated (Figure 1a–f). Not only did PRO-PD correlate with standard ‘motor’ assessments (HY: r = 0.4862, *p* < 0.001; UPDRS: r = 0.4676, *p* = 0.001) it was also correlated with quality of life (PROMIS: r = −0.2631, *p* = 0.081; PDQ-39: r = 0.7335, *p* < 0.001), non-motor symptoms (NMSS, r = 0.7837, *p* < 0.001), and cognitive function (MoCA: r = −0.3358, *p* = 0.026).

Descriptive statistics for each symptom and item-level psychometric characteristics for both datasets are presented in Table 2. Across the large remote-monitoring cohort (*n* = 2612), item distributions demonstrated minimal ceiling effects and generally acceptable skewness, with floor effects exceeding 15% primarily for hallucinations/delusions (37.1%) and nausea (30.6%). Corrected item–total correlations were ≥0.30 for most items, with lower correlations observed for tremor and sense of smell. This pattern closely mirrors findings from the Swedish PRO-PD validation study [15], which similarly reported the lowest item–total correlations for tremor (0.19) and olfaction (0.22), as well as the highest floor effects for hallucinations and nausea. Ceiling effects remained minimal in both datasets (<15% for all items), consistent with the scale’s capacity to capture higher symptom severity. Any minor differences in ceiling proportions between the small in-person sample and the large remote cohort likely reflect sample size and sampling variability rather than mode of administration. The smaller in-person dataset (*n* = 46) demonstrated comparable item-level patterns, with greater variability attributable to sample size. Overall, consistency in item performance across datasets and concordance with prior validation findings support the stability of the PRO-PD item structure across populations and modes of administration.

### 3.1. Internal Consistency

Internal consistency was high for both samples: Cronbach’s α = 0.93 (95% CI: 0.90–0.96) for Small Data and 0.95 (95% CI: 0.947–0.951) for Big Data.

### 3.2. Temporal Stability (Test–Retest Reliability)

Temporal stability was good (ICC = 0.78 overall; 0.89 at 6 months). Item-level ICCs with 95% confidence intervals are presented in Table S5 (Supplementary Materials). Two items (Control of body temperature and Medication side effects) showed very high baseline-to-6-month ICC values (0.984 and 1.000, respectively). These reflect minimal within-person variation over this interval: many participants reported identical or near-identical values at both time points. For medication side effects, ICC = 1.000 with a zero-width confidence interval occurs when residual (within-person) variance is negligible; the bootstrap interval collapses at the ceiling. These values do not indicate a computational error but rather high temporal stability for these specific items over 6 months. Point estimates remain valid for the normative database.

### 3.3. Factor Analysis

To facilitate comparison with the Swedish validation study, a confirmatory factor analysis (CFA) was first conducted using the previously identified eight-factor structure. Model fit indices indicated suboptimal fit (CFI and TLI < 0.90; Table S2). Consequently, exploratory factor analyses were performed, evaluating solutions with four to seven factors. Scree plot inflection and the Kaiser criterion (eigenvalue > 1) supported retention of four factors (Figure S1, Table S3). Parallel analysis suggested five factors. However, we retained four based on interpretability and parsimony. The fifth factor did not yield a conceptually distinct domain, and the four-factor solution demonstrated clearer clinical relevance (Figure S2). Medication side effects, nausea, and dyskinesia were retained in the model despite being primary treatment-related complications rather than intrinsic disease manifestations. In the factor solution, these symptoms loaded most strongly on the autonomic domain, which may suggest these complications manifest through autonomic pathways, as dopaminergic therapies can affect gastrointestinal motility, blood pressure regulation, and other autonomic functions.

Although the 4-factor solution explained a lower proportion (47.6%) of total variance than the previously proposed 8-factor structure (61%) (Table S4), variance explained alone is not a sufficient criterion for model adequacy. The Swedish 8-factor solution was derived using exploratory methods and maximized variance by subdividing correlated symptom domains, whereas confirmatory testing in the present dataset demonstrated suboptimal model fit. The four-factor solution represents a more parsimonious and interpretable structure, prioritizing latent construct validity and overall model fit over variance maximization. This approach aligns with contemporary psychometric standards for patient–reported outcome validation.

### 3.4. PRO-PD in Relation to Demographic and Clinical Characteristics

**Figure 2:**
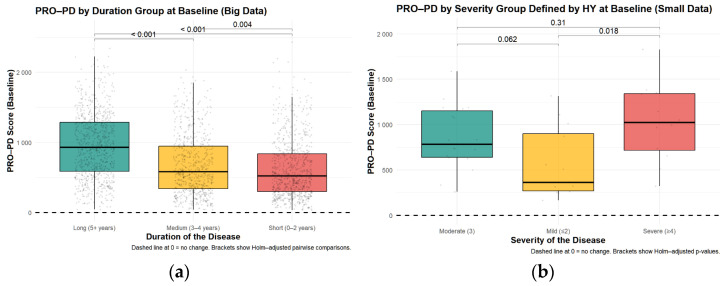
PRO-PD scores with increasing disease duration. (**a**) Large dataset: *n* = 2612; remote patient monitoring, (**b**) Small data: *n* = 45; In-person Parkinson specific evaluation.

Baseline PRO-PD scores were examined across disease duration groups (0–2 years, 3–4 years, and ≥5 years since diagnosis). The large sample size (*n* = 2612) provided substantial statistical power to detect clinically meaningful differences in PRO-PD scores for known clinical groups, supporting the robustness of these comparisons. In the smaller in-person dataset, no significant differences in total PRO-PD scores were observed across duration groups (Kruskal–Wallis χ^2^ = 2.29, *df* = 2, *p* = 0.318). This non-significant finding may reflect limited statistical power (*n* = 46 divided across three groups) rather than a true absence of association. In contrast, in the large remote-monitoring dataset, PRO-PD scores differed significantly by disease duration (Kruskal–Wallis χ^2^ = 251.87, *df* = 2, *p* < 2.2 × 10^−16^). Pairwise comparisons using Wilcoxon rank-sum tests with Holm adjustment confirmed significant differences between all duration groups, with higher PRO-PD scores observed in individuals with longer disease duration. Specifically, comparisons between the ≥5-year group and both the 3–4-year and 0–2-year groups were highly significant (both *p* < 2 × 10^−16^), as was the comparison between the 3–4-year and 0–2-year groups (*p* = 0.0037). These differences are illustrated in Figure 2a,b, which shows a stepwise increase in baseline PRO-PD scores with increasing years since diagnosis.

**Figure 3:**
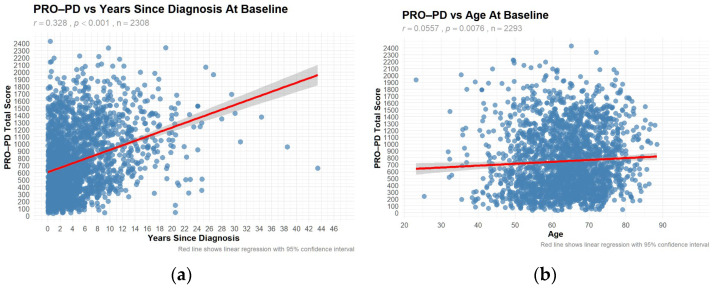
Patient–Reported Outcomes in Parkinson’s Disease (PRO-PD) scores with increasing disease duration and age. High PRO-PD scores represent greater symptom severity. Disease severity increased with (**a**) years since diagnosis, and less so with (**b**) age.

Only the large data demonstrated a significant correlation between PRO-PD and years since diagnosis (r = 0.328, *p* < 0.001) and age (r = 0.0557, *p* = 0.0076) (Figure 3a,b). The correlation with years since diagnosis was also observed in a Swedish study [14], where similar results were obtained.

### 3.5. PRO-PD by Gender at Baseline

**Figure 4:**
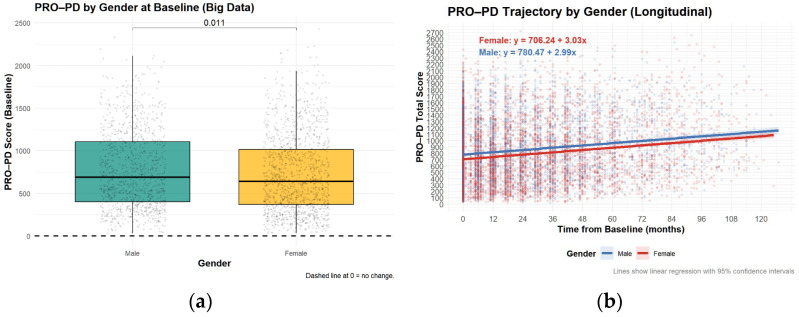
Patient–Reported Outcomes in Parkinson’s Disease (PRO-PD) scores by sex. High PRO-PD scores represent greater symptom severity. (**a**) Symptom severity of sexes at baseline (**b**) Symptom severity of sexes over time.

Contrary to the Swedish study, there was a significant difference in PRO-PD scores between sexes, although the difference was not clinically relevant (Figure 4).

### 3.6. Minimally Clinically Important Difference (MCID)

**Figure 5:**
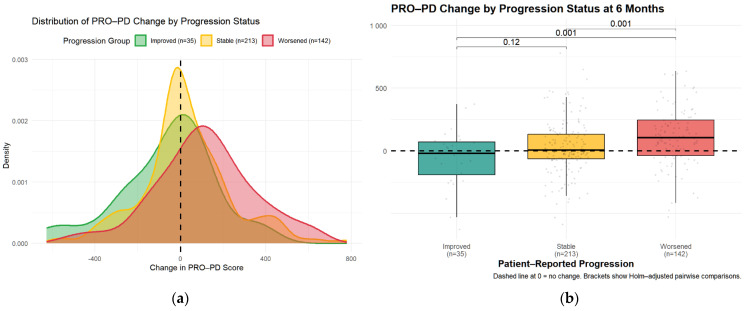
Distribution of Change in PRO-PD Scores by Self-Reported Progression Status at 6 Months. (**a**) Kernel density distributions of PRO-PD change for participants reporting improvement, stability, or worsening. (**b**) Box-and-whisker plots of PRO-PD change with individual observations and Holm-adjusted pairwise comparisons.

Minimal clinically important difference (MCID) was evaluated using an anchor question administered at six months. Of 390 participants, 35 (9%) reported improvement, 142 (36%) reported worsening, and 213 (55%) reported no change. Median PRO-PD change was 7 in the stable group, −20 in the improved group, and +106.5 in the worsened group. Kruskal–Wallis testing demonstrated significant differences between the worsened group and both the stable and improved groups (both *p* = 0.0015), with no significant difference between stable and improved participants (Figure 5). These findings were confirmed using a multinomial regression approach presented later in this section.

**Figure 6:**
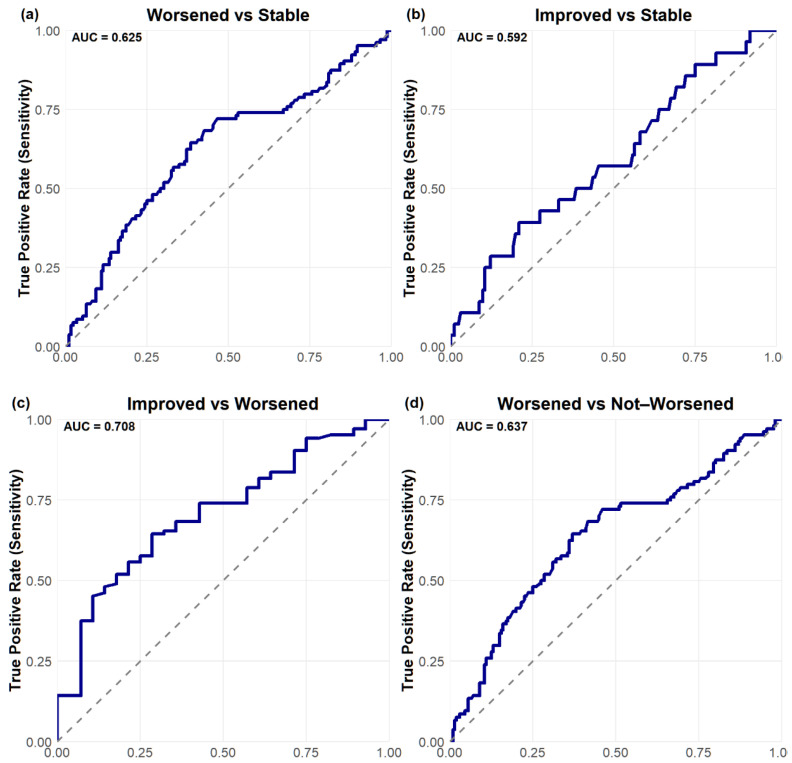
Receiver Operating Characteristic (ROC) curves for change in PRO-PD scores at 6 months. Panels show (**a**) worsened versus stable, (**b**) improved versus stable, (**c**) improved versus worsened, and (**d**) worsened versus not worsened (stable/improved). Axis labels: False Positive Rate (1 − Specificity), True Positive Rate (Sensitivity). The diagonal reference line indicates chance level (AUC = 0.5).

Receiver operating characteristic (ROC) analyses identified a PRO-PD change threshold of +53.5 points distinguishing worsened from stable participants, which was identical for worsened versus not worsened (stable/improved) classifications. This threshold corresponds closely to the Swedish validation study [14], which reported a cutoff of 119 points over a longer follow-up interval, suggesting approximately linear symptom progression over shorter time frames. The MCID for improvement was −78.5 points, indicating a larger magnitude of change required to detect improvement compared with deterioration. Discrimination was modest for improved versus stable participants (AUC = 0.592) and acceptable for improved versus worsened participants (AUC = 0.708). Classification of worsened versus not worsened yielded an AUC of 0.637, with a sensitivity of 0.644 and a specificity of 0.630 (Figure 6. Table 3).

A substantial proportion of participants reporting stable status demonstrated large PRO-PD changes, suggesting potential recall bias or response shift in anchor-based self-assessment. Accordingly, some misclassification may reflect limitations of patient–reported anchors rather than scale performance. Future studies should further refine MCID thresholds using objective clinical anchors (Table 4).

**Figure 7:**
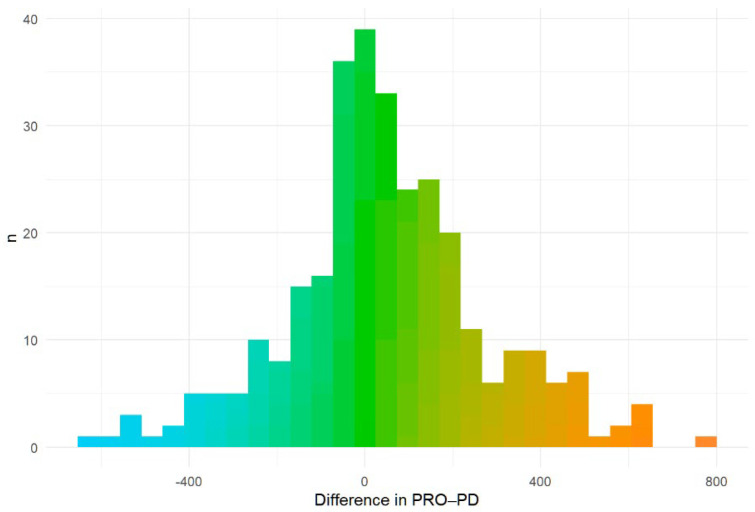
Histogram of differences in PRO-PD at six months from baseline. Colors represent direction and magnitude of change in PRO-PD score, with cooler colors (blue/teal) indicating improvement (negative change), green indicating minimal change, and warmer colors (yellow/orange) indicating worsening (positive change).

Over a six-month period, 213 (54.6%) reported remaining stable (mean change 7 [IQR: −65, 133]), 35 (9.0%) reported improvement (mean change −20.0, [IQR: −192.8, 70], and 142 (36.4%) worsened (mean change: 106.5, [IQR: −38, 246] (Figure 7).

To further evaluate whether a change in PRO-PD score discriminated among self-reported progression categories, a multinomial logistic regression model was fit with progression status (improved, stable, worsened) as the outcome and 6-month change in PRO-PD as the predictor, using the stable group as the reference. Greater increases in PRO-PD score were significantly associated with higher odds of being classified as worsened versus stable (OR = 1.002 per unit increase; *p* = 0.0028). In contrast, increasing PRO-PD change scores were associated with lower odds of being classified as improved versus stable, although this association did not reach conventional statistical significance (OR = 0.998; *p* = 0.055). These findings were concordant with non-parametric analyses, in which overall group differences were significant (Kruskal–Wallis *p* < 0.001), and pairwise comparisons demonstrated significant separation between stable and worsened groups (Holm-adjusted *p* = 0.0015), but weaker discrimination between improved and stable participants. Consistency across analytical approaches supports the construct validity of PRO-PD change scores for differentiating patient-perceived worsening, with more modest sensitivity for detecting improvement over a 6-month interval.

## 4. Discussion

This study provides the first comprehensive psychometric validation of the PRO-PD as a patient–reported outcome measure capable of detecting clinically meaningful change in PD, with relevance to nutrition- and lifestyle-based research. Across two independent datasets, the PRO-PD demonstrated excellent internal consistency, good temporal stability, strong convergent validity with established clinical scales, and robust known-groups validity. Importantly, the scale captured symptom burden across motor and non-motor domains, aligning with contemporary views of PD as a multi-system condition and addressing key limitations of motor-centric outcome measures.

Factor analytic findings support a parsimonious four-domain structure encompassing neurobehavioral, autonomic, motor, and mood/motivation symptoms. Although this structure explained less variance than the previously proposed eight-factor model, it demonstrated superior interpretability and construct validity under confirmatory testing. This trade-off reflects a well-recognized psychometric principle: variance maximization alone does not ensure meaningful latent structure. The four-factor structure aligns conceptually with neuropathological staging of PD, whereby Braak stages 1–2 involve olfactory and lower brainstem regions (early non-motor features), later spreading to midbrain dopaminergic regions and eventually to limbic and neocortical areas. Consistent with this framework, several items loading on the autonomic and sleep-related domains (e.g., constipation, dysautonomia, REM sleep behavior disorder) have been associated with early brainstem involvement, whereas the motor factor reflects dysfunction of the nigrostriatal dopaminergic system that becomes prominent with substantia nigra degeneration. In contrast, symptoms clustering within cognitive and neuropsychiatric domains may reflect later limbic and cortical involvement. Although factor analysis captures patterns of symptom co-occurrence rather than temporal disease stages, the observed clustering of clinically meaningful symptom domains is broadly consistent with the spatial progression of pathology described in Braak staging and supports the biological plausibility of the identified factor structure. This four-factor structure also has potential clinical implications. These clinically recognizable symptom clusters may facilitate more structured assessment of multidimensional symptom burden. Grouping symptoms into these domains may help clinicians and patients interpret patient–reported outcomes more intuitively, track domain-specific changes over time, and identify areas of disproportionate symptom impact.

Anchor-based analyses established asymmetric MCID thresholds, with smaller changes required to detect worsening than improvement, a pattern consistent with prior PD studies and patient–reported outcome research more broadly. The MVP study used a three-category anchor (improved, stable, worsened), enabling asymmetric MCID thresholds for both improvement and worsening, whereas the Swedish study compared only deteriorated versus unchanged. The present study also explicitly examined patterns of misclassification in anchor-based MCID estimation, attributing discordance between PRO-PD change and self-reported status to limitations of patient–reported anchors rather than scale performance. The moderate discrimination observed for improvement likely reflects both biological asymmetry and limitations of global recall anchors, rather than inadequate scale sensitivity. The strong concordance between non-parametric, multinomial, and ROC-based methods supports the robustness of the identified thresholds and provides practical benchmarks for interpreting longitudinal change in interventional studies.

Floor and ceiling effects were examined to assess the dynamic range of the PRO-PD items. Ceiling effects were minimal across items in both datasets, indicating that responses rarely clustered at the maximum score and that it retains sensitivity for detecting worsening symptoms. Floor effects were more common for several items, which is expected in a heterogeneous population where many symptoms are absent in a subset of individuals. Overall, the observed distribution supports the ability of the PRO-PD to capture a broad spectrum of symptom severity without substantial saturation at the extremes. These characteristics are particularly important for outcome measures intended for longitudinal monitoring.

### 4.1. Limitations

This study has several limitations. PD remains a clinicopathologic diagnosis, with definitive confirmation possible only at autopsy. Although in-person examination, specialist history, and biomarker confirmation would have enhanced diagnostic certainty, such assessments were not feasible within the remote design. Diagnostic misclassification cannot be excluded, and some participants’ diagnoses may evolve over time. The remote, patient–reported nature limits independent verification of diagnoses and symptom severity. Findings should be interpreted considering reliance on participant-reported diagnosis and symptom experience.

The convergent validity sample (*n* = 46) was small and drawn from a single clinical trial, limiting generalizability. Hoehn and Yahr staging was unavailable in the MVP cohort (remote design), and known-groups validity by disease severity was therefore evaluated only in the small in-person sample (*n* = 46), limiting statistical power for that comparison. The anchor-based MCID analyses relied on patient recall of global change over 6 months, which may be subject to recall bias or response shift, particularly among individuals reporting stable symptoms despite large PRO-PD changes. The number of participants classified as improved was relatively small (*n* = 35), which may limit statistical power for detecting improvement and contribute to the more modest AUC values observed for improved versus stable comparisons. The moderate AUC values for discriminating improved versus stable participants suggest that detecting improvement may be more challenging than detecting worsening. Estimates of the MCID for improvement should be interpreted cautiously and will require confirmation in future studies with larger numbers of improving participants.

While these findings support the utility of the PRO-PD total score for capturing overall symptom burden at the population level, its validity and clinical usefulness for individual patient management remain to be established. Future studies are needed to determine whether PRO-PD scores can assist clinicians in goal setting, identifying patients at higher risk for adverse outcomes, or monitoring response to therapeutic interventions over time. The four-factor structure identified in the analysis may provide an additional framework for interpreting symptom patterns within the overall PRO-PD score, but its role in guiding clinical decision-making will require further evaluation.

Table S1 provides a structured comparison of PRO-PD’s strengths and limitations relative to the MDS-UPDRS. Notably, while PRO-PD offers advantages in remote monitoring and patient-centeredness, it lacks the objective motor assessments and regulatory acceptance of established clinician-rated scales.

### 4.2. Conclusions

The PRO-PD demonstrates strong psychometric performance, sensitivity to clinically meaningful change, and minimal floor effects in early PD. Its rapid completion time (<10 min), remote accessibility without requiring trained administrators, and responsiveness to patient-perceived symptom burden position it as a complementary outcome measure for intervention trials where traditional motor-centric scales may lack sensitivity or feasibility. These properties support its use as a patient–reported outcome measure in intervention studies, including those evaluating nutrition, lifestyle, or pharmacologic approaches, where patient-perceived symptom burden is of interest. Although the validation itself is not directly related to nutrition, it is a necessary prerequisite for further research, including studies building diet scores with PRO-PD as the response variable. No analyses in this manuscript were designed to test the effects of diet, nutraceuticals, or lifestyle factors on PD symptoms, and no causal inferences should be drawn regarding such exposures. Future work should include wearable sensor comparison, external validation, and prospective intervention studies to confirm the scale’s responsiveness and clinical utility.

## 5. Intellectual Property

LK Mischley owns the copyright of the Patient–Reported Outcomes in Parkinson’s Disease (PRO-PD) scale, which was made freely available for these research projects.

## Figures and Tables

**Table 1:** Participant characteristics. Small data were obtained from baseline values from a clinical trial; large data were obtained from an observational study employing remote patient monitoring. Continuous variables are presented as median [Q1, Q3] unless otherwise specified; categorical variables are presented as n (%).

Sample Characteristics at Baseline				
	**Small Data** **(In-Person Clinical Trial Data, MDS Diagnosed)**		**Large Data** **(Remote Patient Monitoring, Patient–Reported)**	
	N = 46	(n)	N = 2612	(n)
Gender:		45		2600
Female	22 (48.9%)		1479 (56.9%)	
Male	23 (51.1%)		1120 (43.1%)	
Non-binary			1 (0.1%)	
Age (years)	64.0 [56.5; 68.5]	35	63.6 (9.4)	2556
Years Since Diagnosis	3.0 [2.0; 4.0]	41	3.3 [1.3; 6.7]	2612
PRO-PD Total Score	741 [499; 1109]	45	664 [381; 1067]	2389
UPDRS Total Score	66.5 [61.2; 74.0]	46		
PROMIS Global Composite Score	33.0 [29.2; 35.0]	46		
PDQ-39 Total Score	17.3 [10.9; 21.2]	45		
Hoehn & Yahr Stage	3.0 [2.0; 4.0]	45		
MoCA Score	28.0 [26.0; 29.0]	44		
NMS Total Score	33.5 [20.2; 48.8]	42		
Race:		45		
White	44 (97.8%)			
Asian/Pacific Islander	1 (2.2%)			

**Table 2:** Descriptive statistics and item analysis.

Small In-Person Data Set (*n* = 46, 34 Items, No Restless Legs)							Large Remote Patient Data Monitoring Data Set (*n* = 2612, 35 Items)					
PRO-PD Item	Mean	SD	Skewness	Floor (%)	Ceiling (%)	Corrected Item-Total Correlation	Mean	SD	Skewness	Floor (%)	Ceiling (%)	Corrected Item-Total Correlation
Slowness	31.2	24.4	0.72	4.4	2.2	0.617	29.3	22.7	0.57	5.4	0.1	0.692
Constipation	24.1	22.7	0.80	15.6	2.2	0.267	22.9	23.3	1.04	15.1	0.3	0.512
Walking	26.8	23	0.54	8.9	2.2	0.734	21.8	19.9	0.99	10.1	0	0.714
Freezing	6.8	12.1	2.15	35.6	2.2	0.309	12.4	19.4	1.89	30.6	0.1	0.568
Falling	8.8	11.6	1.62	22.2	2.2	0.365	14.1	19.7	1.74	25.4	0.1	0.605
Rising from a seated position	18	18.7	0.83	20	2.2	0.616	20.2	19.9	0.92	15.9	0	0.705
Dressing/eating/grooming	15.8	17.2	1.14	15.6	2.2	0.607	17.6	18.7	1.26	16.6	0.1	0.698
Motivation/initiative	22.4	20.9	0.98	6.7	2.2	0.708	25.4	23.7	0.77	11.8	0.1	0.663
Handwriting/typing	40.6	25.4	0.10	2.2	2.2	0.603	36.5	26.2	0.42	6.1	0.8	0.569
Depression	14.9	11.8	0.52	4.4	2.2	0.585	18.7	20.1	1.54	14.4	0.2	0.616
Loss of interest	19.5	17.7	0.96	13.3	2.2	0.652	20.9	22.8	1.14	16	0.2	0.673
Anxiety	23.7	22.4	1.23	6.7	2.2	0.555	24.8	24.3	1.06	11.3	0.5	0.603
Fatigue	35.2	24.3	0.40	4.4	2.2	0.753	35	25.3	0.4	5.1	0.5	0.746
Daytime sleepiness	34	26.2	0.34	6.7	2.2	0.813	29.2	24.2	0.63	7.8	0.1	0.685
Dyskinesia	13.2	19.1	1.46	24.4	2.2	0.463	15.8	22.3	1.44	27.8	0.1	0.490
Tremor	40.6	25	−0.26	2.2	4.4	0.138	27.2	21.8	0.78	5.3	0.1	0.357
Balance	20	16.3	1.04	8.9	2.2	0.705	23.6	21.3	1.16	9	0.3	0.687
Control of body temperature	20.1	22.2	1.28	17.8	2.2	0.522	21.9	22.7	1.01	14.1	0.4	0.470
Dizzy on standing	16	19.4	1.40	15.6	2.2	0.584	15.9	20.2	1.61	20.1	0.1	0.610
Visual disturbance	16.7	21.1	1.83	11.1	2.2	0.527	15	20.6	1.62	24.7	0	0.637
Insomnia	28	27.5	0.83	6.7	2.2	0.404	29.2	27.3	0.73	11.5	0.6	0.563
Acting out dreams	19.8	23.3	1.18	13.3	2.2	0.134	22.9	26	1.06	19.6	0.4	0.517
Restless legs							19.5	24.2	1.31	20.9	0.3	0.549
Muscle cramping/pain/aching	25.5	26.4	1.08	4.4	2.2	0.622	29.4	25.2	0.75	7.2	0.2	0.651
Speech	27.3	20.3	0.36	4.4	2.2	0.725	21.5	21.1	0.89	15	0	0.657
Drooling	20.6	21.7	1.04	15.6	2.2	0.091	20.9	23.3	0.88	21.1	0	0.489
Stooped posture	30.1	26.1	0.72	8.9	2.2	0.522	24.7	21.1	0.66	11.1	0	0.626
Memory/forgetfulness	32.7	20.5	−0.26	4.4	2.2	0.729	28.2	21.7	0.48	7.5	0	0.672
Comprehension	21.5	18.8	0.59	13.3	2.2	0.721	17.8	19.7	1.25	17.2	0	0.664
Sense of smell	44.1	35.8	0.02	11.1	4.4	0.265	45.3	33	0.04	8.6	2.3	0.317
Medication side effects	24.5	23.6	0.76	6.7	2.2	0.526	19.5	24.3	1.24	20.5	0.4	0.490
Sexual dysfunction	32.3	29	0.62	6.7	2.2	0.498	35.9	31.3	0.52	10.9	2.1	0.497
Urinary symptoms	29.4	26.5	0.52	8.9	2.2	0.653	31.5	27	0.5	10.2	0.5	0.583
Hallucinations/delusions	7.6	14.1	2.79	24.4	2.2	0.363	7.1	13.8	2.9	37.1	0	0.479
Nausea	13.8	20.6	1.93	22.2	2.2	0.517	10	16.8	2.37	30.6	0	0.483

PRO-PD: Patient–Reported Outcomes in Parkinson’s Disease; SD: Standard deviation.

**Table 3:** Receiver Operating Characteristic (ROC) Performance Metrics: PRO-PD change score classification performance. AUC: Area Under the Curve, Optimal threshold based on Youden’s index. Higher PRO-PD change scores indicate worsening.

Performance Metrics					
Comparison	AUC	Optimal Threshold	Sensitivity	Specificity	Accuracy
Worsened vs. Stable	0.625	53.5	0.644	0.616	0.627
Improved vs. Stable	0.592	−78.5	0.393	0.791	0.735
Improved vs. Worsened	0.708	53.5	0.644	0.714	0.659
Worsened vs. Stable/Improved	0.637	53.5	0.644	0.63	0.635

Note: AUC = Area Under the Curve. Optimal threshold based on Youden’s index.

**Table 4:** Clinical interpretation of PRO-PD change threshold for detecting symptom worsening.

Clinical Meaning of PRO-PD Change Thresholds		
Metric	Value	Clinical Meaning
Sensitivity (catch worsening)	0.644	64.4% of worsened patients were correctly identified
Specificity (avoid false alarms)	0.630	63% of stable patients were correctly identified
PPV (if flagged, truly worsened?)	0.475	47.5% of flagged patients are truly worsened
NPV (if not flagged, truly stable?)	0.773	77.3% of non-flagged patients are truly stable
Accuracy	0.635	63.5% overall correct classification
False Positive Rate	0.370	37% of stable were wrongly flagged

## Data Availability

Data will be made available to qualified researchers upon request, with approval of the University.

## Supplementary Materials

The following supporting information can be downloaded at: https://www.mdpi.com/article/10.3390/nu18071118/s1, Table S1: Measurement orientation and research applications of Parkinson’s disease outcome measures; Table S2: Application of Swedish eight-factor model to the English dataset; Table S3: Exploratory factor analysis: four-factor solution (MVP cohort, *n* = 2612); Table S4: Variance Explained by 4-Factor Solution (MVP cohort, *n* = 2612); Table S5: ICC with 95% CI (Big data, 35 items) for all time points and baseline and 6 months together; Figure S1: Scree plot of eigenvalues for PRO-PD items (MVP cohort, *n* = 2612). Dashed line indicates Kaiser criterion (ei-genvalue = 1). Four factors had eigenvalues exceeding 1, supporting retention of the 4-factor solution; Figure S2: Scree plot with parallel analysis for determination of factor retention. Observed eigenvalues (blue) are com-pared with simulated random data (red). Dashed line indicates Kaiser criterion (eigenvalue = 1). Parallel analysis suggested five factors; the four-factor solution was retained based on interpretability and parsimony.
