# Supplementary material for: Validation of a Patient–Reported Outcome Measure in Parkinson’s Disease
Source: Nutrients. 2026 Mar 31;18(7):1118. doi: 10.3390/nu18071118 (PMC13074223; doi:10.3390/nu18071118)
Supplement: Supplementary file 1 [file nutrients-18-01118-s001.zip › nutrients-4159166-Supplementary.pdf]

SUPPLEMENTARY MATERIALS for **Validation of a Patient-Reported Outcome Measure in Parkinson's Disease**

Table S1. Measurement orientation and research applications of Parkinson's disease outcome measures.

Outcome measurement frameworks in Parkinson's disease: clinician-rated and patient-reported approaches		
**Dimension**	**PRO-PD (Patient-Reported Outcomes in Parkinson's Disease)**	**MDS-UPDRS (Movement Disorder Society–Unified Parkinson's Disease Rating Scale)**
**Primary measurement goal**	Quantification of subjective symptom burden across motor and non-motor domains	Standardized assessment of motor signs, activities of daily living, and complications
**Conceptual anchor**	Within-person change and lived symptom severity	Between-patient comparison and examiner-verified impairment
**Rater**	Person with Parkinson's disease (self-report)	Trained clinician or examiner
**Assessment context**	Remote or in-clinic; self-administered	In-clinic, structured examination
**Domains emphasized**	Motor + non-motor, including cognitive, autonomic, and psycho-emotional symptoms	Motor examination (Part III), patient experience (Parts I–II), motor complications (Part IV)
**Sensitivity to early or non-motor change**	High sensitivity to non-motor symptoms and subtle perceived changes	Moderate; optimized for observable motor change
**Temporal resolution**	Designed to detect gradual change over months while minimizing day-to-day fluctuation	Optimized for clinically observable change across defined assessment intervals
**Floor and ceiling effects**	Minimal floor effects in early disease; continuous scoring	Reduced granularity in very early disease; ordinal scaling
**Measurement granularity**	Continuous (0-100), enabling fine-grained detection of small changes	Ordinal (0-4 per item), optimized for clinically distinuishable motor states
**Minimal clinically important change**	Anchor-based MCID established using patient global impression of change	Clinically meaningful change well characterized in trial contexts
**Inter-rater variability**	Not applicable	Present; mitigated through rater training and certification
**Participant burden**	Low; no travel or examiner required	Moderate; requires clinic visit and trained personnel
**Trial infrastructure requirements**	Well suited to decentralized and pragmatic designs	Requires centralized clinical infrastructure
**Utility for lifestyle and behavioral research**	High sensitivity to patient-perceived effects of diet, supplements, sleep, and activity	Limited sensitivity to lifestyle effects unless reflected in motor examination
**Utility for pharmacologic trials**	Complementary endpoint reflecting patient-perceived benefit	Primary regulatory endpoint with established precedent
**Regulatory familiarity**	Emerging	Extensive; gold standard in pivotal trials
**Primary strengths**	Captures patient-centered symptom burden and longitudinal change with high feasibility	Provides objective, standardized motor assessment with regulatory acceptance
**Primary limitations**	Subject to self-report bias and perception-dependent variability	Resource-intensive; less sensitive to early and non-motor change

Table S2. Application of Swedish eight-factor model to the English dataset.

Confirmatory Factor Analysis Fit Indices for Swedish 8-Factor Model

Fit Index	Value	Acceptable Fit	Good Fit	Interpretation
Chi-square (χ^2)	1492.23	—	—	—
Degrees of Freedom (df)	532	—	—	—
p-value	< 0.001	> 0.05	> 0.05	Poor fit
CFI	0.807	> 0.90	> 0.95	Below acceptable
TLI	0.784	> 0.90	> 0.95	Below acceptable
RMSEA	0.083	< 0.08	< 0.06	Acceptable
RMSEA 90% CI	[0.078, 0.088]	—	—	—
SRMR	0.079	< 0.08	< 0.05	Acceptable

[1] CFI: Comparative Fit Index, TLI: Tucker-Lewis Index, RMSEA: Root Mean Square Error of Approximation, SRMR: Standardized Root Mean Square Residual.

Table S3. Exploratory factor analysis: four-factor solution (MVP cohort, n=2612)

	Factor Loadings			
**PRO-PD Item**	**Factor 1 Neurobehavioral**	**Factor 2 Autonomic**	**Factor 3 Motor**	**Factor 4 Mood/ Motivation**
Slowness		0.30	0.70	
Constipation	0.32			
Walking			0.75	

Freezing			0.62	
Falling	0.30		0.61	
Rising from seated position			0.73	
Dressing/eating/grooming			0.70	
Motivation/initiative			0.34	0.69
Handwriting/typing	0.30		0.51	
Depression				0.68
Loss of interest				0.79
Anxiety	0.30	0.33		0.54
Fatigue		0.48	0.43	0.43
Daytime sleepiness		0.41	0.35	0.38
Dyskinesia		0.41	0.30	
Tremor		0.30		
Balance			0.71	
Control of body temperature		0.53		
Dizzy on standing	0.37	0.40		
Visual disturbance	0.46	0.32	0.34	
Insomnia		0.42		
Acting out dreams (RBD)	0.42	0.31		
Restless legs	0.30	0.40		
Muscle cramping/pain		0.56	0.33	
Speech	0.49		0.48	
Drooling	0.4		0.35	
Stooped posture	0.44		0.47	
Memory/forgetfulness	0.6			0.40
Comprehension	0.53			0.44
Sense of smell	0.37			
Medication side effects		0.74		
Sexual dysfunction	0.39			
Urinary symptoms	0.41		0.31	
Hallucinations/delusions	0.44			
Nausea		0.53		

Note:

Only loadings ≥ 0.30 shown.

Table S4. Variance Explained by 4-Factor Solution (MVP cohort, n=2612).

Factor	Eigenvalue	% Variance	Cumulative %
Factor 1: Neurobehavioral	5.69	16.3%	16.3%
Factor 2: Autonomic	3.77	10.8%	27.0%
Factor 3: Motor	3.68	10.5%	37.6%
Factor 4: Mood/Motivation	3.51	10%	47.6%
**Total**	—	**47.6%**	—

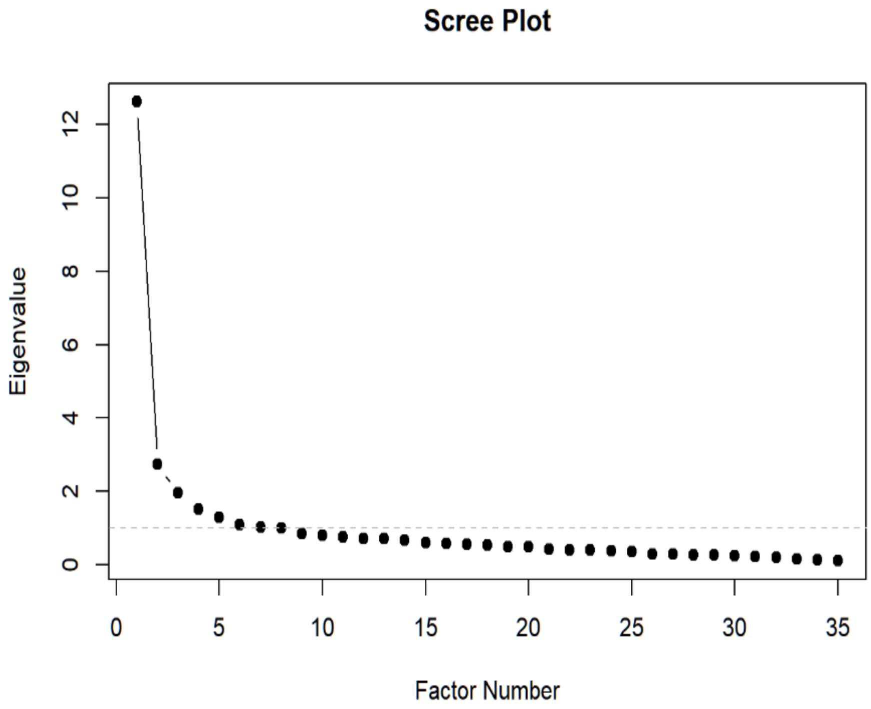

Figure S1. Scree plot of eigenvalues for PRO-PD items (MVP cohort, n=2612). Dashed line indicates Kaiser criterion (eigenvalue = 1). Four factors had eigenvalues exceeding 1, supporting retention of the 4-factor solution.

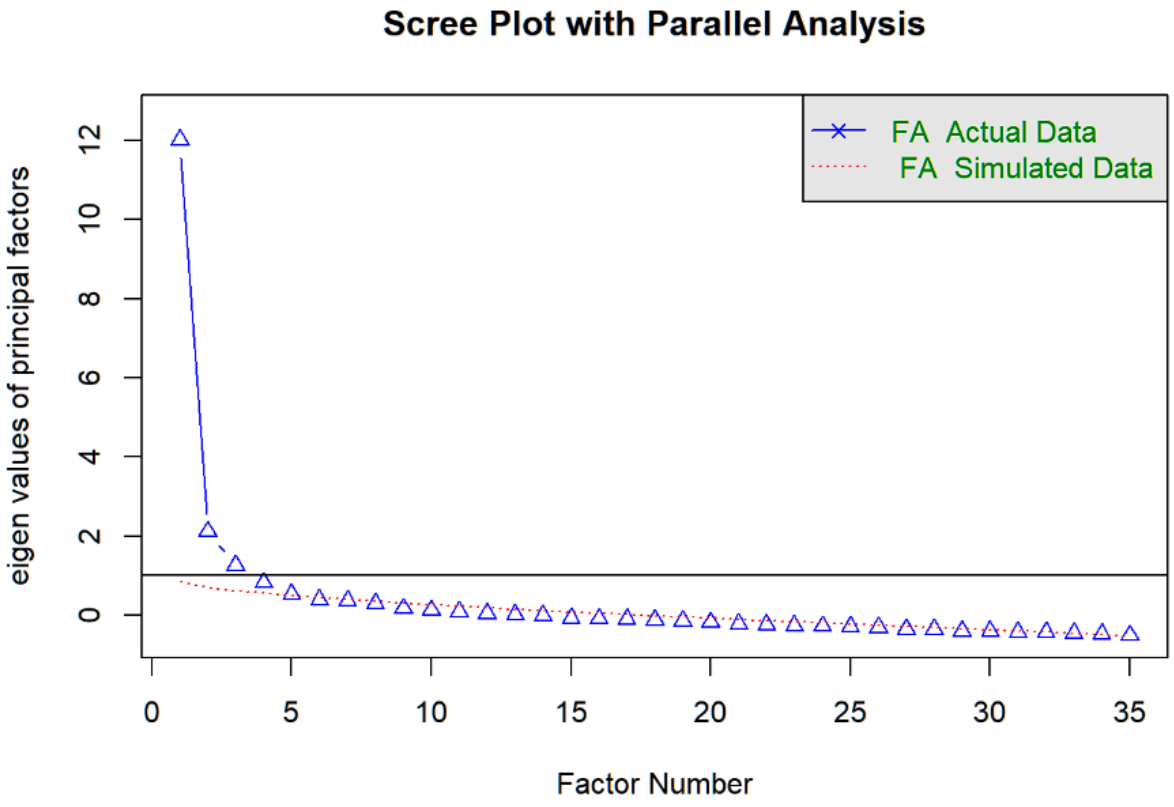

Figure S2. Scree plot with parallel analysis for determination of factor retention. Observed eigenvalues (blue) are compared with simulated random data (red). Dashed line indicates Kaiser criterion (eigenvalue = 1). Parallel analysis suggested five factors; the four-factor solution was retained based on interpretability and parsimony.

Table S5. ICC with 95%CI (Big data, 35 items) for all time points and baseline and 6 months together.

PRO-PD Item	all time points<br>ICC (95% CI)	baseline and 6 months<br>ICC (95% CI)
Slowness	0.604 (0.587–0.620)	0.643 (0.597–0.682)
Constipation	0.669 (0.650–0.683)	0.720 (0.680–0.752)
Walking	0.632 (0.616–0.649)	0.784 (0.753–0.806)
Freezing	0.630 (0.613–0.648)	0.825 (0.799–0.849)
Falling	0.670 (0.654–0.684)	0.819 (0.792–0.844)
Rising from seated position	0.682 (0.669–0.695)	0.808 (0.778–0.838)
Dressing/eating/grooming	0.659 (0.647–0.677)	0.781 (0.746–0.810)

**Motivation/initiative**	0.601 (0.583–0.616)	0.716 (0.679–0.747)
**Handwriting/typing**	0.679 (0.666–0.693)	0.784 (0.758–0.817)
**Depression**	0.660 (0.644–0.676)	0.759 (0.730–0.794)
**Loss of interest**	0.649 (0.628–0.665)	0.719 (0.687–0.753)
**Anxiety**	0.649 (0.630–0.667)	0.709 (0.657–0.738)
**Fatigue**	0.646 (0.629–0.662)	0.763 (0.735–0.792)
**Daytime sleepiness**	0.625 (0.606–0.643)	0.760 (0.731–0.792)
**Dyskinesia**	0.580 (0.557–0.596)	0.797 (0.767–0.824)
**Tremor**	0.640 (0.620–0.657)	0.755 (0.731–0.782)
**Balance**	0.678 (0.663–0.692)	0.795 (0.762–0.817)
**Control of body temperature**	0.730 (0.619–0.813)	0.984 (0.927–1.000)
**Dizzy on standing**	0.625 (0.608–0.638)	0.731 (0.689–0.766)
**Visual disturbance**	0.592 (0.573–0.609)	0.706 (0.666–0.743)
**Insomnia**	0.640 (0.624–0.656)	0.736 (0.699–0.768)
**Acting out dreams (RBD)**	0.672 (0.661–0.687)	0.775 (0.749–0.803)
**Restless legs**	0.639 (0.625–0.653)	0.728 (0.695–0.765)
**Muscle cramping/pain**	0.615 (0.598–0.635)	0.768 (0.740–0.797)
**Speech**	0.681 (0.668–0.696)	0.746 (0.716–0.774)
**Drooling**	0.653 (0.640–0.668)	0.772 (0.741–0.798)
**Stooped posture**	0.678 (0.662–0.694)	0.778 (0.752–0.810)
**Memory/forgetfulness**	0.687 (0.671–0.701)	0.783 (0.754–0.817)
**Comprehension**	0.667 (0.655–0.684)	0.708 (0.660–0.747)
**Sense of smell**	0.813 (0.803–0.822)	0.874 (0.849–0.891)

**Medication side effects**	0.772 (0.667–0.858)	1.000 (1.000–1.000)
**Sexual dysfunction**	0.683 (0.668–0.695)	0.728 (0.688–0.762)
**Urinary symptoms**	0.663 (0.646–0.677)	0.749 (0.717–0.785)
**Hallucinations/delusions**	0.563 (0.539–0.581)	0.592 (0.537–0.634)
**Nausea**	0.515 (0.489–0.532)	0.621 (0.560–0.679)
